# Comparative Transcriptome Analysis of Gene Responses of Salt-Tolerant and Salt-Sensitive Watermelon Cultivars’ Roots to Salt Stress

**DOI:** 10.3390/plants14071013

**Published:** 2025-03-24

**Authors:** Peng Liu, Chao Gao, Shuai Li, Xudong Wang, Yumei Dong, Chongqi Wang, Zigao Jiao, Jianlei Sun

**Affiliations:** Shandong Key Laboratory of Bulk Open-Field Vegetable Breeding, Ministry of Agriculture and Rural Affairs Key Laboratory of Huang Huai Protected Horticulture Engineering, Institute of Vegetables, Shandong Academy of Agricultural Sciences, Jinan 250100, China; saaslp2023@163.com (P.L.);

**Keywords:** watermelon (*Citrullus lanatus* L.) root, salinity tolerance, physiological phenotype, transcriptome

## Abstract

Salt stress, as a significant adverse consequence of global climate change, severely restricts the yield and quality of watermelon. In this study, salt-tolerant cultivar T23 and salt-sensitive cultivar B2 were subjected to a 200 mM NaCl treatment (0 h, 6 h, 24 h, 48 h, and 168 h) at the three-leaf stage, and the adaptation mechanisms of the watermelon roots to salt stress were systematically investigated at the phenotypic, physiological, and gene transcription levels. Phenotypic observations revealed that salt stress inhibited seedling growth, caused leaf curling, and induced root yellowing, with the damage being significantly more severe in B2 than in T23. Compared with B2, the activities of superoxide dismutase (SOD) were increased by −7.13%, 169.15%, 34.95%, 84.87%, and 39.87% under NaCl treatment at 0 h, 6 h, 24 h, 48 h, and 168 h, respectively. Compared to the 0 h NaCl treatment, the proline content in B2 increased by 4.25%, 14.39%, and 110.00% at 24 h, 48 h, and 168 h of NaCl treatment, respectively, while T23 showed increases of 93.74%, 177.55%, and 380.56% at the corresponding time points. The provided physiological data demonstrate that T23 exhibits superior antioxidant and osmoregulatory abilities relative to B2. The transcriptome analysis identified differentially expressed genes (DEGs) between the two cultivars under salt stress, with T23 showing the highest number of DEGs at 6 h, while B2 exhibited a significant increase in DEGs at 168 h. Gene Ontology (GO) and Kyoto Encyclopedia of Genes and Genomes (KEGG) enrichment analysis revealed that metabolic pathways such as plant hormone signal transduction, terpenoid biosynthesis, mitogen-activated protein kinase (MAPK) signaling pathways, transporter activity, and transcription regulator activity play important roles in the salt stress response. Furthermore, yeast overexpression experiments preliminarily validated the critical roles of the tonoplast dicarboxylate transporter gene *ClCG01G010280* and the NAC transcription factor gene *ClCG05G024110* in salt stress tolerance. This study provides new molecular insights into the salt tolerance mechanism of watermelon and offers potential genetic resources for breeding salt-tolerant varieties.

## 1. Introduction

Soil salinization poses a significant constraint on plant growth and development, particularly affecting moderately salt-sensitive crops such as watermelon (*Citrullus lanatus* L.). When subjected to elevated salinity conditions, watermelon plants demonstrate distinct physiological and biochemical adaptations [1]. The primary mechanism of salt stress involves the disruption of osmotic regulation, which consequently impairs water absorption and nutrient homeostasis [2]. This results in stunted growth, wilting, and leaf chlorosis, which are common visible symptoms in watermelon plants under salt stress [3]. Salt stress also affects watermelon fruit quality and yield [4,5,6]. High salinity levels can lead to smaller fruit size, reduced sugar content, and altered fruit composition [6]. The accumulation of sodium ions (Na^+^) and chloride ions (Cl^−^) in the soil competes with essential nutrients like potassium (K^+^) and calcium (Ca^2+^), further exacerbating nutrient deficiencies and impairing fruit development [4,7,8,9]. Therefore, understanding the molecular mechanism of salt tolerance in watermelon and exploring key salt-tolerance genes is important for breeding robust, salt-tolerant watermelon varieties.

Plants have developed various protective mechanisms in response to salt stress, such as signal transduction, ion homeostasis regulation, metabolic pathway adjustments, and the activation of resistance genes [10,11,12]. Plant hormone signaling and MAPK signaling pathways are crucial in responding to abiotic stress, regulating physiological changes and gene expression [13,14,15]. Salt stress defense relies on numerous genes involved in two signaling pathways. Terpenoid biosynthesis, thiamine metabolism, and carbon metabolism are crucial for plant resistance to salt stress [16,17]. Multiple transcription factors (TFs) regulate gene expression in response to salt stress, among which salt-responsive transcription factors are activated through multiple signaling pathways in plants. Evidence indicates that transcription factors (TFs) are differentially regulated under salt stress, involving NAC, MYB, and WRKY gene families. This suggests that TFs play crucial roles in modulating the acclimation response to salt stress [18,19,20,21,22,23].

At the cellular level, salt stress induces oxidative damage by generating reactive oxygen species (ROS), which can damage membranes, proteins, and DNA [24]. Watermelon plants activate antioxidant defense mechanisms, including enzymes like superoxide dismutase (SOD), peroxidase (POD), and catalase (CAT), to mitigate oxidative stress [25]. Furthermore, plants improve their stress resistance by accumulating osmotic regulators like proline and soluble sugars [26,27]. The accumulation of these substances within cells effectively reduces the osmotic potential of the cells, thereby maintaining cell turgor pressure and ensuring normal physiological functions. Specifically, proline, a crucial osmotic regulator, plays a significant role in stabilizing protein structures and cell membranes, while also scavenging free radicals to mitigate oxidative damage [28,29,30]. Soluble sugars like sucrose and glucose elevate the cytoplasmic concentration, reduce water potential, and enhance water absorption and retention [31]. Plants utilize these mechanisms to maintain the cellular water balance under high-salt conditions, thereby ensuring normal metabolic activities and growth [32]. However, prolonged exposure to high salinity frequently overwhelms these defenses, resulting in cellular damage and decreased photosynthetic efficiency.

Advances in sequencing technology have enabled researchers to utilize transcriptome sequencing data for investigating metabolic pathways involved in plant responses to salt stress, as well as for identifying potential salt-tolerant genes. Liu et al. speculated through transcriptome sequencing that watermelon seedlings mainly cope with salt stress by regulating amino acid and carbohydrate metabolism pathways [33]. Meanwhile, the sugar or energy pathways, nitrogen metabolism, photosynthesis, and hormone pathways in response to salt stress has been given special attention [34,35]. Furthermore, a candidate gene that mediates salt tolerance, *Cla97C09G174040*, was identified through transcriptome profiling analysis [34]. Salt-tolerant candidate genes have been identified in rice [36], tomato [37], cucumber [38], and soybean [39] using transcriptome sequencing technology. These studies highlight RNA sequencing technology’s substantial potential in identifying critical metabolic pathways related to salt tolerance and discovering candidate genes involved in plant salt tolerance.

The root system, which interfaces directly with the soil environment, serves as the primary site for detecting salt stress. Soil salts are absorbed into the plant through the roots, making the root system the ‘first line of defense’ against salt stress. High-salinity conditions reduce soil water potential, thereby hindering water absorption by the roots. Simultaneously, the excessive accumulation of salt ions (e.g., Na^+^ and Cl^−^) disrupts the uptake of essential nutrients (e.g., K^+^, Ca^2+^) [34]. Examining the physiological and molecular responses of roots to salt stress is essential for comprehending how plants maintain water and nutrient balance. Under salt stress, roots employ multiple mechanisms to regulate the absorption, transport, and distribution of salt ions. For instance, root cells can mitigate the toxic effects of salt ions through processes such as ion efflux, compartmentalization (sequestering Na^+^ into vacuoles), and the selective uptake of K^+^ [40]. Investigating the physiological, biochemical, and molecular responses of roots to salt stress enhances our understanding of plant adaptation mechanisms.

The cultivation area of watermelon in China exceeds 1 million hectares, making it a major fruit crop. However, watermelon has a relatively weak root system and is sensitive to salt stress. Therefore, mitigating the impact of salt damage on watermelon has become an urgent issue. Investigating salt tolerance mechanisms and identifying salt-tolerant genes are crucial for developing new salt-resistant varieties and fostering the sustainable growth of the watermelon industry. In this study, salt-tolerant and salt-sensitive watermelon varieties were used as experimental materials. First, phenotypic changes and physiological indicators related to salt tolerance were measured in watermelon seedlings treated with 200 mM NaCl over different time periods to determine their tolerance to salt stress. Subsequently, RNA sequencing analysis was performed on the roots of the two varieties to analyze transcriptional differences under salt stress and to identify candidate genes associated with salt tolerance in watermelon. This research provides a scientific basis for breeding new salt-tolerant varieties.

## 2. Materials and Methods

### 2.1. Plant Materials and Experiment Design

The salt-tolerant watermelon T23 (the plant grows strongly, with thick skin, yellow flesh, and large seeds) and salt-sensitive watermelon B2 (the plant has weak growth, thin skin, yellow flesh color, and small seeds) were used in this study. Uniform seeds were soaked in a constant-temperature and -humidity chamber (29 °C, 90% humidity) for 2 h. After removal and light drying, seeds were placed in an 18 cm culture dish on double-layered filter paper, covered with a top layer of filter paper, and 10 mL of distilled water was added. After germination, seeds showing visible whitening were transplanted into trays filled with Half-strength Hoagland nutrient solution. The composition of the Hoagland nutrient solution is detailed in Table S1. The nutrient solution was renewed every 3 days. When the seedlings reached the three-leaf stage, the Hoagland nutrient solution was replaced with nutrient solution containing 200 mM NaCl. Seedlings having similar growth vigor were selected and treated for 0 h, 6 h, 24 h, 48 h, and 168 h with 200 mM NaCl. Thress seedlings were randomly selected from each replicate of each treatment, and a total of 9 roots of the watermelon seedlings were clipped and collected for transcriptome and physiological analyses at the corresponding treatment time. All samples were frozen in liquid nitrogen and stored at −80 °C. Three biological replicates were performed for each sample in this study.

### 2.2. Measurement of Physiological Parameters

The activities of SOD, POD, and CAT were determined using the nitroblue tetrazole (NBT) photochemical reduction method, the guaiacol method, and the hydrogen peroxide (H_2_O_2_) ammonium molybdate catalytic decomposition method [41]. The content of proline, soluble protein, and soluble sugars was determined using the sulfosalicylic acid-indanone colorimetric method, the Coomassie Brilliant Blue (G250) colorimetric method, and the anthrone-sulfuric acid colorimetry method [27]. The content of determination of H_2_O_2_, superoxide anion (O^2−^), and Malondialdehyde (MDA) was carried out using the titanium sulfate (titanium chloride) colorimetric method, hydroxylamine hydrochloride p-aminobenzene sulfonic acid-α-naphthylamine colorimetric method, and the thiobarbituric acid colorimetric method [31]. All physiological parameters were determined by Wuhan ProNets Testing Technology Co., Ltd., Wuhan, China.

### 2.3. RNA Sequencing and Data Analysis

Total RNA from the 30 samples was extracted using TRIzol reagent (Invitrogen, Carlsbad, CA, USA) according to the manufacturer’s instructions. The quality, quantity, and RNA integrity (RIN) number were measured using a Nanodrop ND 1000 spectrophotometer and an Agilent Technologies 2100 Bioanalyzer (Agilent, Santa Clara, CA, USA). The cDNA sequencing libraries were constructed using an NEB Next Ultra RNA Library Prep Kit for Illumina (NEB, San Diego, CA, USA) following the manufacturer’s recommendations, and index codes were added to attribute sequences to each sample. The double-stranded cDNA was purified, washed in EB buffer for end repair, added with single-nucleotide A (Adenine), and ligated with sequencing adaptors. All libraries were sequenced on an Illumina HiSeq X-ten platform by Beijing Biomarker Technologies Co., Ltd. (Beijing, China). B2 and T23 plants exposed to salt stress for 0 h, 6 h, 24 h, 48 h, and 168 h were named B0/T0, B6/T6, B24/T24, B48/T48, and B168/T168, respectively.

Before downstream analyses, we filtered low-quality or adaptor-polluted reads and those with a high content of unknown base (N) reads using internal software. Clean data (clean reads) were obtained by removing low-quality reads and reads containing adapter and poly-N from the raw data and Q20, Q30, GC-content and sequence duplication level of the clean data were calculated. After filtering, the remaining reads were stored in a FASTQ format for downstream analysis. Clean reads from every sample were mapped to the reference Watermelon Charleston Gray genome v2.5 (http://cucurbitgenomics.org). Raw counts of genes were determined using feature counts [42]. Differentially expressed genes (DEGs) between two samples DESeq2 (EBSeq) with |log_2_fold change| ≥ 1 and a false discovery rate (FDR) < 0.05 were considered significant expression differences [43]. DEG functions were annotated using the Kyoto Encyclopedia of Genes and Genomes (KEGG) and Gene Ontology (GO) databases [44]. The comparisons B0 vs. B6, B0 vs. B24, B0 vs. B48, and B0 vs. B168 in the transcriptome comparison analysis of B2 are referenced as B0–B6, B0–B24, B0–B48, and B0–B168, respectively. Similarly, comparisons of T0 vs. T6, T0 vs. T24, T0 vs. T48, and T0 vs. T168 are referenced as T0–T6, T0–T24, T0–T48, and T0–T168, respectively.

### 2.4. Fluorescent Quantitative Real-Time PCR (qRT-PCR) Verification

To verify the reliability of RNA sequencing results, 12 DEGs were randomly selected from the transcriptome data for qRT-PCR. Based on the coding gene sequences, the RT-qPCR primers were designed using Primer Premier 6.0 software, and β-actin was selected as the internal control gene (Table S2). Total RNA extraction and detection, cDNA preparation and detection, and real-time quantitative RT-PCR detection and analysis were performed as described by Harshitha et al. [45]. Three independent biological replicates and three technological replicates were used for each sample.

### 2.5. Yeast Constructs

To construct the yeast (*Saccharomyces cerevisiae*) overexpression vectors, the coding sequences of watermelon genes, *ClCG03G001690*, *ClCG10G009260*, *ClCG09G022760*, *ClCG09G002220*, *ClCG01G010280*, *ClCG05G024110*, *ClCG05G009670*, and *ClCG05G024910*, were cloned separately into the pRS-416-GFP vector. The coding sequences of the genes were amplified from watermelon cDNA with specific primers (Supplementary Table S2) and then inserted into the SPE1 site on pRS-416-GFP using the infusion cloning kit (Catalog no. 011614; Clontech, Mountain View, CA, USA). The sequence insertions were confirmed through SANGER sequencing and then used to investigate salt stress tolerance in yeast [46].

### 2.6. Tolerance Assay and Growth Curve

The final pRS-416-GFP vectors cultured in URA medium were diluted until the OD600 value was 0.1, and were incubated again until the OD600 reached 0.3. The cell culture was then diluted five-fold and treated with 1 M NaCl, and was incubated at 28 °C for five days [47]. No treatment was added for the control. The photos were taken after five days of incubation.

### 2.7. Data Processing and Visualization

SPSS 25.0 software was used for variance analysis using Duncan’s multiple comparison method [41]. The bar diagram was plotted using Microsoft Excel 2011. Venn diagrams, scatter diagrams, and heat maps were drawn using BMKCloud tools (https://www.biocloud.net) [36].

## 3. Results

### 3.1. Phenotypic Changes in Watermelon Seedlings of Two Varieties Under Salt Stress

**Figure 1:**
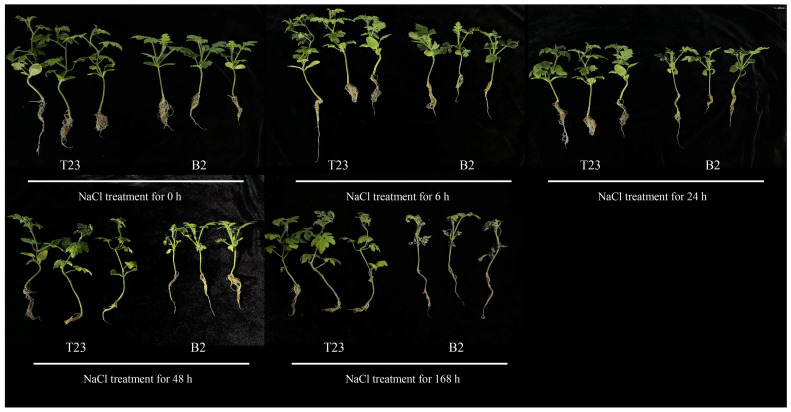
Phenotype of watermelon seedlings exposed to 200 mM NaCl for different stages.

Two watermelon varieties, the salt-tolerant T23 and the salt-sensitive B2, were treated with a 200 mM NaCl solution (Figure 1). At 6 h of salt treatment, the B2 seedlings exhibited significant signs of stress: their cotyledons dehydrated and curled, true leaves wilted, and roots turned yellow. In contrast, the T23 plants showed no notable changes. At 24 h of salt treatment, the symptoms in the B2 seedlings worsened, with increased dehydration of the cotyledons, further wilting of the true leaves, and more pronounced root yellowing. The T23 plants began to show slight yellowing in their root systems. At 48 h of salt treatment, the B2 seedlings displayed intensified dehydration and yellowing in both cotyledons and lower true leaves, while the T23 plants exhibited yellowing in their cotyledons and a more pronounced yellowing in their root systems. At 168 h of salt treatment, the B2 plants’ root systems turned black, and some plants died. Meanwhile, the T23 plants showed dried-up cotyledons and yellowing in the leaves and roots, but no mortality was observed. Throughout the salt treatment period, the B2 variety consistently suffered more severe damage compared to the T23 variety.

### 3.2. Analysis of Physiological Parameters Under Salt Stress

**Figure 2:**
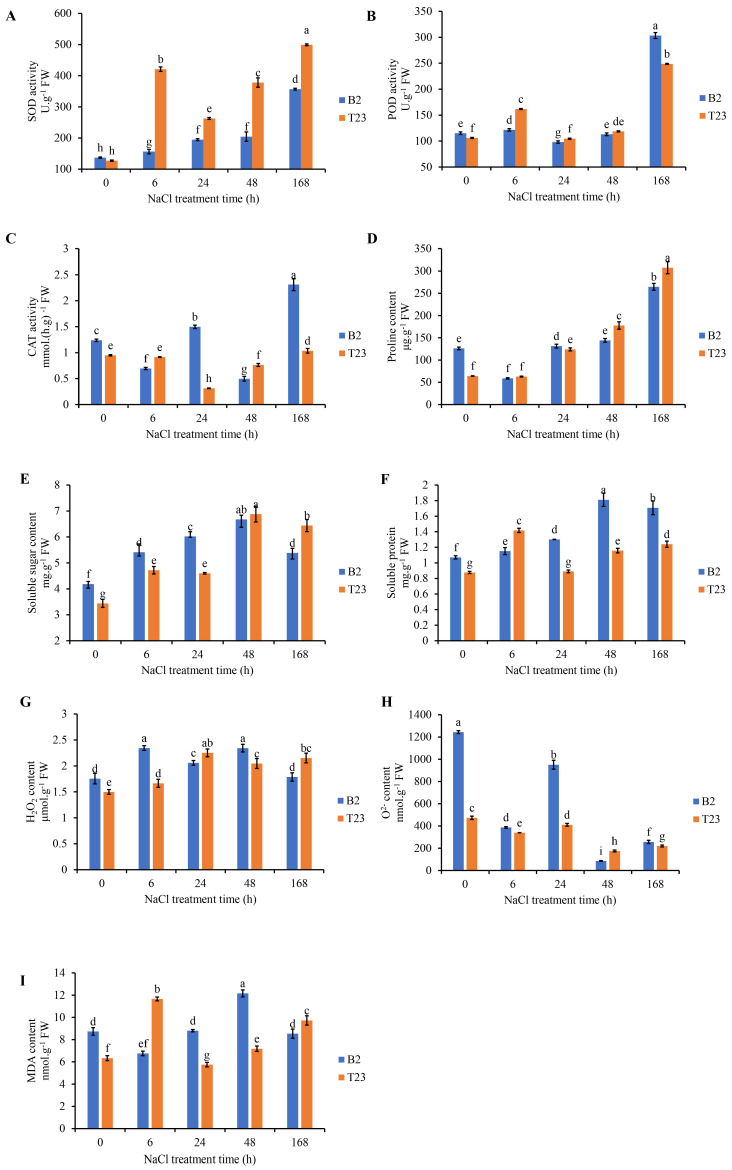
Variation in physiological indexes of watermelon plants under salt stress. (**A**) SOD activity, (**B**) POD activity, (**C**) CAT activity, (**D**) proline content, (**E**) soluble sugar content, (**F**) soluble protein content, (**G**) H_2_O_2_ content, (**H**) O^2−^ content, and (**I**) MDA content. Error bars represent standard deviation among means for three different samples. Different letters on bars indicate statistically significant differences (*p* < 0.05) among different varieties or different salt treatment time points.

Changes in stress resistance physiology of watermelon roots exposed to 200 mM NaCl were evaluated. Under salt exposure, the activity of SOD significantly increased in both varieties. Compared with B2, the activities of SOD were increased by −7.13%, 169.15%, 34.95%, and 84.87% at 0, 6, 24, 48, and 168 h of salt treatment, respectively (Figure 2A). Compared with no salt treatment, the POD activity of T23 significantly increased at 6, 24, and 168 h of salt treatment by 52.59%, 11.97%, and 134.57%, respectively. While the POD activity of B2 significantly increased by 5.74% and 163.73% at 6 and 168 h, respectively (Figure 2B). Compared with no salt treatment, the CAT activity of B2 showed fluctuating changes, while the CAT activity of T23 first decreased and then increased. At 24 and 168 h of salt treatment, the CAT activity of B2 was significantly increased by 21.75% and 87.71% (Figure 2C).

The proline content of the two varieties decreased after 6 h of salt treatment, then increased, and reached its peak at 168 h of salt treatment. At 24, 48, and 168 h of salt treatment, the proline content of T23 was significantly higher, by 93.74%, 177.55%, and 380.56%, than that at 0 h of salt treatment, respectively. Meanwhile, the proline content of B2 was significantly increased by 110.00% at 168 h of salt treatment, respectively (Figure 2D). The soluble sugar content was significantly increased under salt exposure in B2 and T23. At 6, 24, 48, and 168 h of salt treatment, the soluble sugar content of T23 was significantly increased by 38.73%, 35.31%, 102.35%, and 89.36% compared to that at 0 h of salt treatment, respectively. Meanwhile, the H_2_O_2_ content of B2 was significantly increased by 28.80%, 43.34%, 58.91%, and 28.20% at 6, 24, 48, and 168 h of salt treatment, respectively (Figure 2E). The soluble protein content of both varieties significantly increased after the salt treatment. At 6, 24, 48, and 168 h of salt treatment, the soluble sugar content of T23 was significantly increased by 61.07%, 1.25%, 31.56%, and 40.90% compared to that at 0 h of salt treatment, respectively. Meanwhile, the H_2_O_2_ content of B2 was significantly increased by 7.64%, 21.61%, 69.19%, and 59.61% at 6, 24, 48, and 168 h of salt treatment, respectively (Figure 2F). The H_2_O_2_ content of both varieties significantly increased after salt treatment. At 6, 24, 48, and 168 h of salt treatment, the H_2_O_2_ content of T23 was significantly increased by 10.93%, 50.05%, 36.39%, and 43.48% compared to that at 0 h of salt treatment, respectively. Meanwhile, the H_2_O_2_ content of B2 was significantly increased by 33.18%, 16.93%, 33.10%, and 1.53% at 6, 24, 48, and 168 h of salt treatment, respectively (Figure 2G). After salt treatment, the O^2−^ content of both varieties significantly decreased. At 0, 6, 24, and 168 h of salt treatment, the O^2−^ content in T23 was significantly decreased by 61.92%, 12.20%, 56.80%, and 14.20% compared to that in B2 (Figure 2H). The MDA content of B2 and T23 first decreased and then increased, while that of T23 first increased and then decreased (Figure 2I).

### 3.3. Evaluation of RNA-Seq Data Quality

Total RNA sequencing was conducted on 30 samples exposed to 200 mM NaCl for durations of 0 h, 6 h, 24 h, 48 h, and 168 h. A total of 185.87 Gb of clean data was obtained. The Q30 quality score for all 30 libraries exceeded 93.90%, with the GC content ranging between 43.82% and 45.35% (Table S3). The mapping readings of each sample were mapped to the designated reference genome, with mapping efficiencies ranging from 83.57% to 96.40% (Table S3). These results indicate that the accuracy of the experimental data is high and suitable for subsequent bioinformatics analysis.

### 3.4. Differential Expression Gene Analysis

To explore the molecular mechanism of salt tolerance in watermelon roots, DEGs in plants under salt stress at different time periods were studied. The criteria for screening DEGs are fold change ≥ 2 and FDR < 0.05. Compared with salt treatment at 0 h, 1191 DEGs (435 upregulated and 756 downregulated), 2731 DEGs (838 upregulated and 1893 downregulated), 2076 DEGs (581 upregulated and 1495 downregulated), and 7020 DEGs (2753 upregulated and 4267 downregulated) were identified in B2 at 6, 24, 48, and 168 h, respectively. Meanwhile, 2607 DEGs (657 upregulated and 1950 downregulated), 1846 DEGs (461 upregulated and 1385 downregulated), 392 DEGs (157 upregulated and 235 downregulated), and 291 DEGs (173 upregulated and 118 downregulated) were identified in T23 at 6, 24, 48, and 168 h, respectively (Table S4). In the 6 h sample collection, T23 had the highest number of DEGs. Conversely, B2 exhibited the highest number of DEGs in the 168 h sample collection. This indicates that there are differences in the response patterns of these two varieties to salt stress. During the entire salt treatment period, the number of upregulated DEGs in both varieties was less than that of the downregulated DEGs (Table S4).

**Figure 3:**
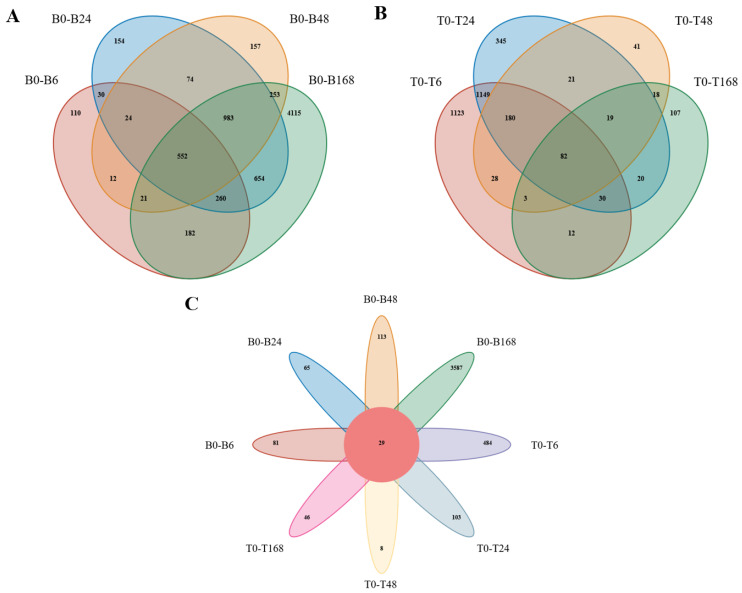
Venn diagrams of DEGs in B2 and T23. (**A**) B0–B6, B0–B24, B0–B48, and B0–B168 comparisons for B2; (**B**) T0–T6, T0–T24, T0–T48, and T0–T168 comparisons for T23; and (**C**) three different compared groups for B2 and T23.

We mapped the DEGs of the two plants in a Venn diagram (Figure 3A,B). For B2, there were a total of 552 DEG intersections for B0–B6, B0–B24, B0–B48, and B0–B168 (Figure 3A). And for the T23, there were a total of 82 DEG intersections for T0-T6, T0-T24, T0-T48, and T0-T168 (Figure 3B). B2 had more unique DEGs than did T23. These eight groups shared 29 DEGs (Figure 3C). A total of 29 DEGs appeared in all eight comparisons, suggesting that this group may contain key genes that respond to salt stress (Figure 3C).

### 3.5. Transcriptome Data Verified by qRT-PCR

**Figure 4:**
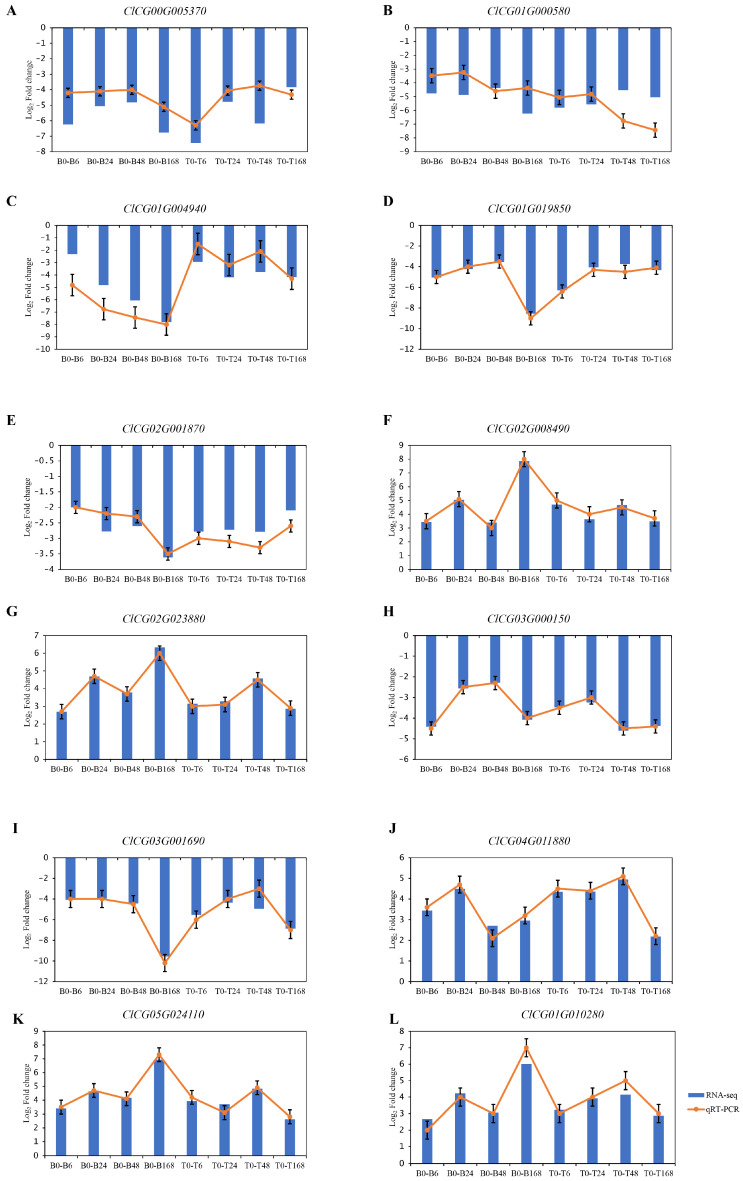
Comparison analysis of qRT-PCR and RNA-seq for 12 genes. (**A**) *ClCG00G005370*, (**B**) *ClCG01G000580*, (**C**) *ClCG01G004940*, (**D**) *ClCG01G019850*, (**E**) *ClCG02G001870*, (**F**) *ClCG02G008490*, (**G**) *ClCG02G023880*, (**H**) *ClCG03G000150*, (**I**) *ClCG03G001690*, (**J**) *ClCG04G011880*, (**K**) *ClCG05G024110*, (**L**) *ClCG01G010280*. The gene expression level of RNA-seq is shown as Log_2_(fold change) and the fold-change is based on FPKM values of the drought stress group relative to the control group. The gene expression level of qRT-PCR is shown as Log_2_ (fold change = 2^−ΔΔCt^).

To verify the reliability of the transcriptome sequencing data, 12 DEGs were randomly selected for qRT-PCR analysis (Table S2). The trends for up-regulated and down-regulated expression were consistent between the RNA-seq and qRT-PCR for all 12 genes (Figure 4). The trend in gene expression changes detected by qRT-PCR is consistent with the results of the RNA-seq. These results confirm that a similar expression pattern of DEGs was detected by qRT-PCR and RNA-seq.

### 3.6. GO Enrichment Analysis

**Figure 5:**
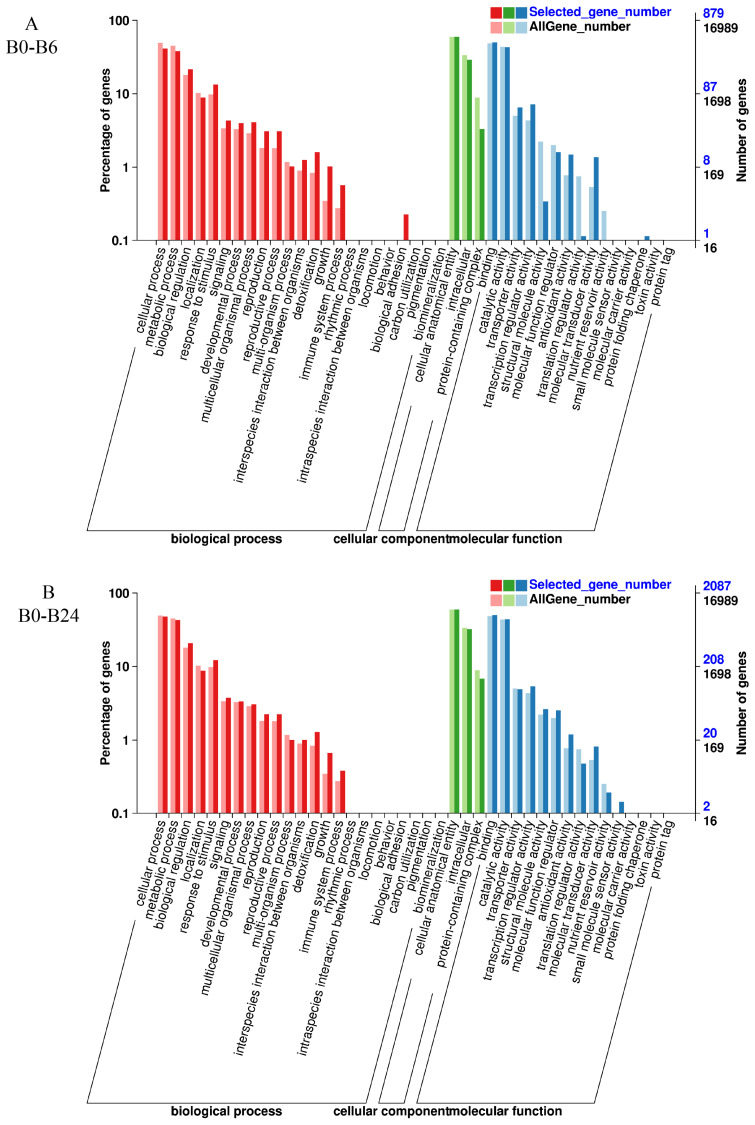

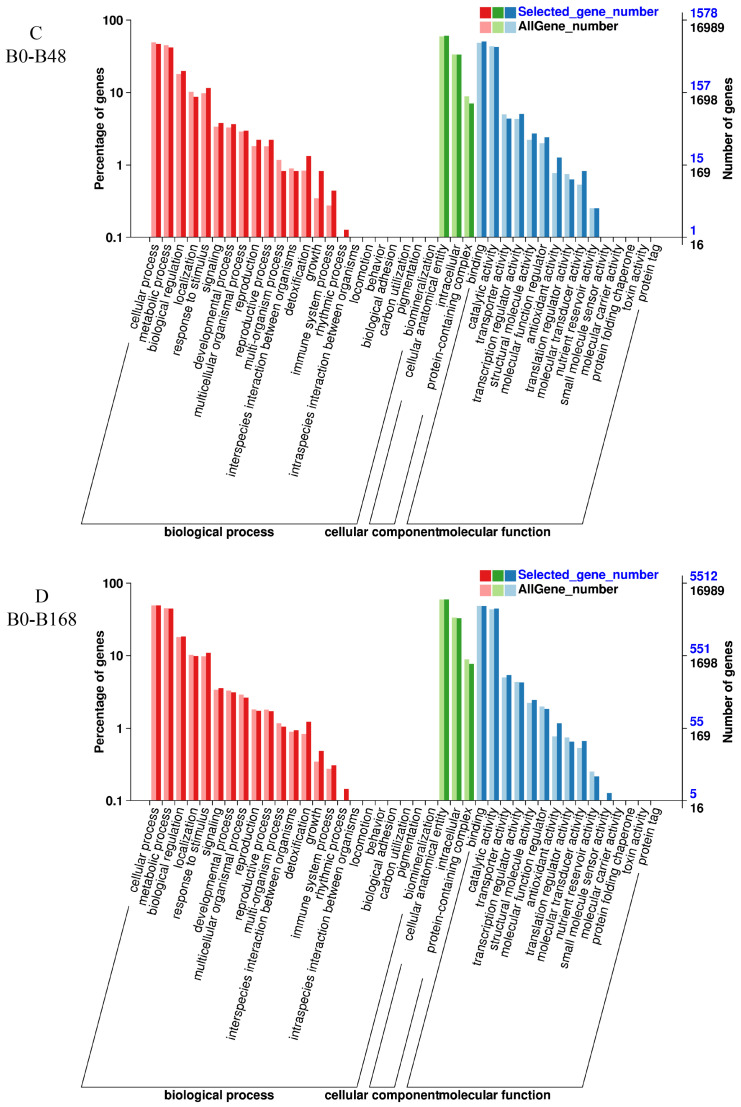
GO enrichment terms in B2 exposed to salt stress for 6 h (**A**), 24 h (**B**), 48 h (**C**), and 168 h (**D**).

Subsequently, we conducted GO analysis on the DEGs of two varieties with different salt treatment times. To explore specific pathways with GO enrichment, the top 10 GO terms were analyzed for biological process (BP), cellular component (CC), and molecular function (MF) components based on q-value < 0.05 (Figure 5). For BP, cellular process, metabolic process, biological regulation, localization, response to stimulus and signaling were present in the four groups of B2 (Figure 5). For CC, cellular anatomical entity and intracellular and protein-containing complex were present in the four groups of B2 (Figure 5). For MF, binding, catalytic activity, transporter activity, transcription regulator activity, and antioxidant activity were shared in the four groups of B2 (Figure 5).

**Figure 6:**
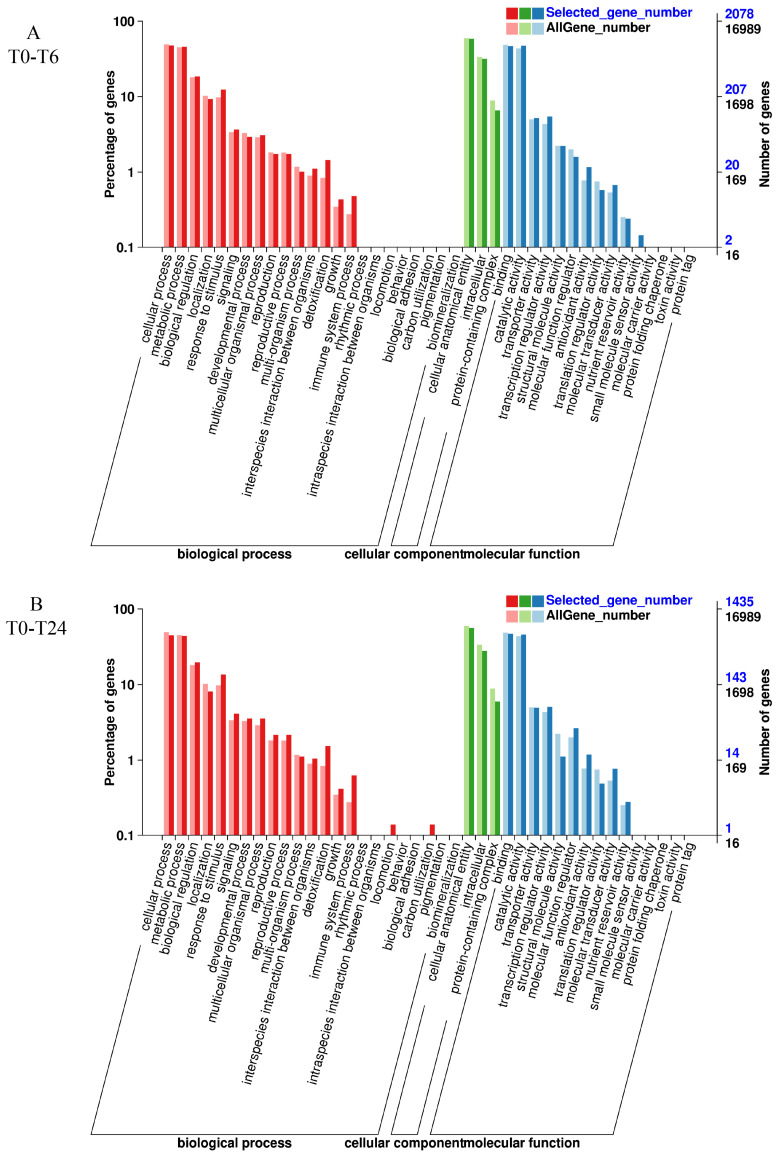

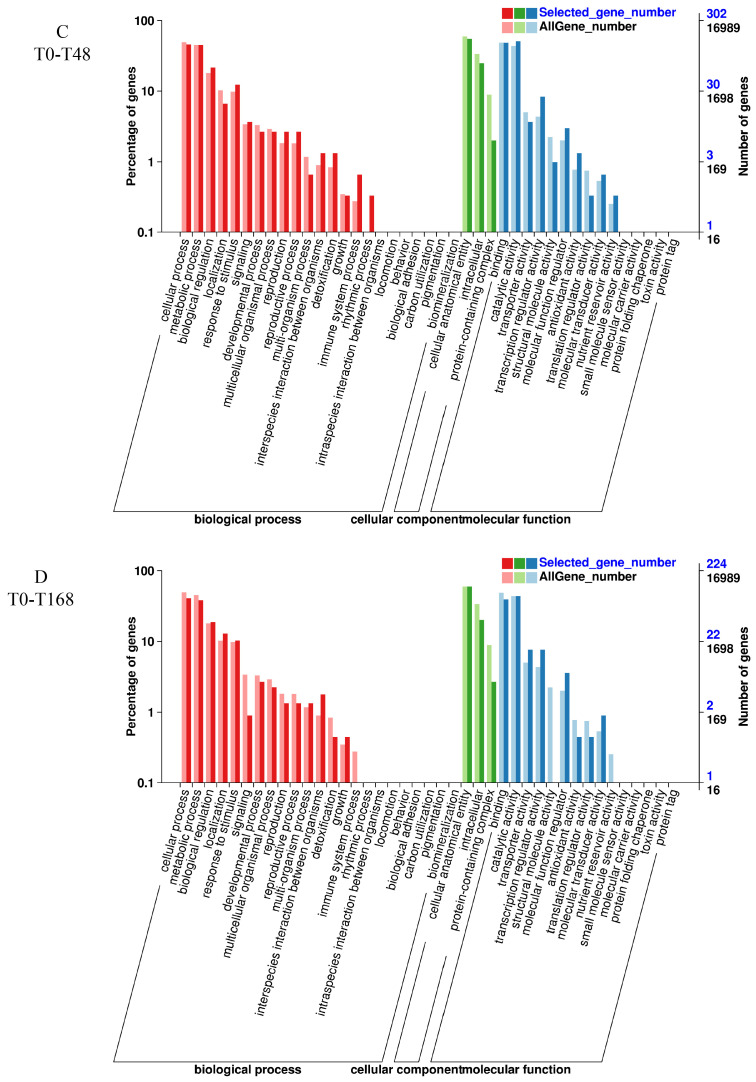
GO enrichment terms in T23 exposed to salt stress for 6 h (**A**), 24 h (**B**), 48 h (**C**), and 168 h (**D**).

The results for T23 were similar to those for B2: cellular process, metabolic process, biological regulation, localization, and response to stimulus and signaling were present in the four groups of T23 (Figure 6). Cellular anatomical entity and intracellular and protein-containing complex were present in the four groups of T23 for CC (Figure 6). And binding, catalytic activity, transporter activity, and transcription regulator activity were shared in the four groups of T23 (Figure 6).

### 3.7. KEGG Enrichment Analysis of Differentially Expressed Genes

**Figure 7:**
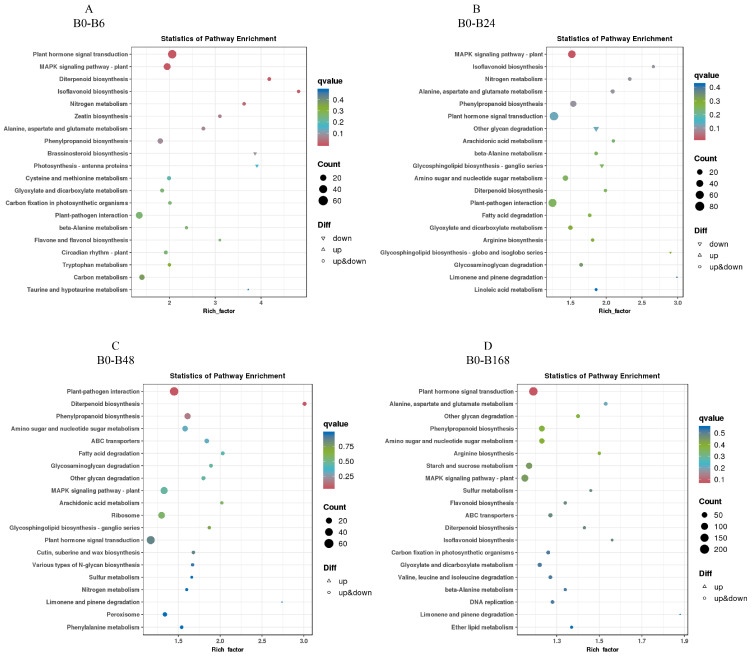
KEGG enrichment pathways in B2 exposed to salt stress for 6 h (**A**), 24 h (**B**), 48 h (**C**), and 168 h (**D**).

To clarify the metabolic pathways of watermelon in response to salt stress, we conducted a KEGG enrichment analysis. For B2, plant hormone signal transduction, MAPK signaling pathway—plant and diterpenoid biosynthesis; MAPK signaling pathway—plant, isoflavonoid biosynthesis, and nitrogen metabolism; plant–pathogen interaction, diterpenoid biosynthesis, and phenylpropanoid biosynthesis; and plant hormone signal transduction, alanine, aspartate, and glutamate metabolism, and other glycan degradation were enriched at 6, 24, 48, and 168 h of salt treatment, respectively. And hormone signal transduction, diterpenoid biosynthesis, plant–pathogen interaction, and MAPK signaling pathway—plant were enriched in four groups together (Figure 7).

**Figure 8:**
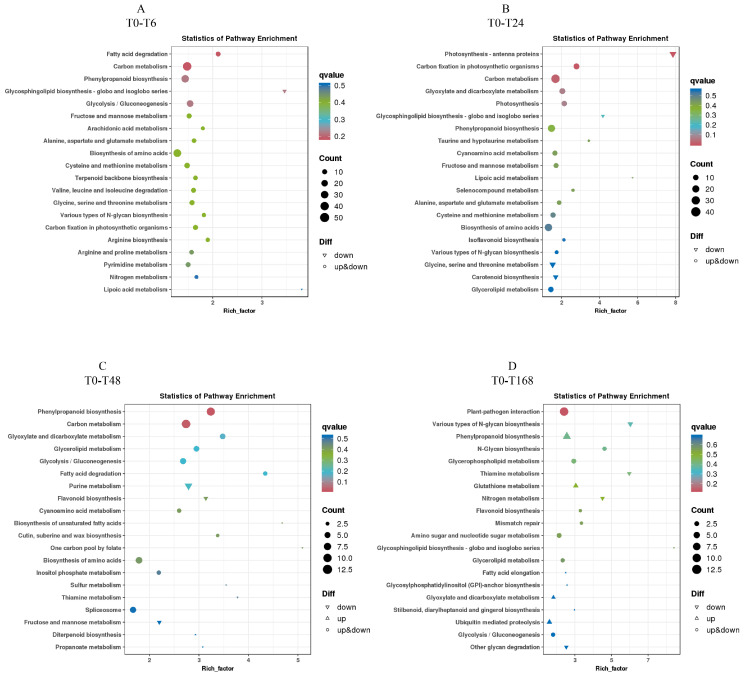
KEGG enrichment pathways in T23 exposed to salt stress for 6 h (**A**), 24 h (**B**), 48 h (**C**), and 168 h (**D**).

For T23, fatty acid degradation, carbon metabolism, and phenylpropanoid biosynthesis; photosynthesis antenna proteins, carbon fixation in photosynthetic organisms, and carbon metabolism; phenylpropanoid biosynthesis, carbon metabolism, and glyoxylate and dicarboxylate metabolism; and plant–pathogen interaction, various types of n-glycan biosynthesis, and phenylpropanoid biosynthesis were enriched at 6, 24, 48, and 168 h of salt treatment, respectively. And thiamine metabolism and carbon metabolism were enriched together (Figure 8).

These results indicate that different genotypes of watermelon have different ways of resisting salt stress, and plant hormone signal transduction, diterpenoid biosynthesis, plant–pathogen interaction, MAPK signaling pathway—plant, thiamine metabolism, and carbon metabolism play a role in watermelon’s resistance to salt stress.

### 3.8. Exploration and Preliminary Verification of Salt-Tolerant Candidate Genes

**Figure 9:**
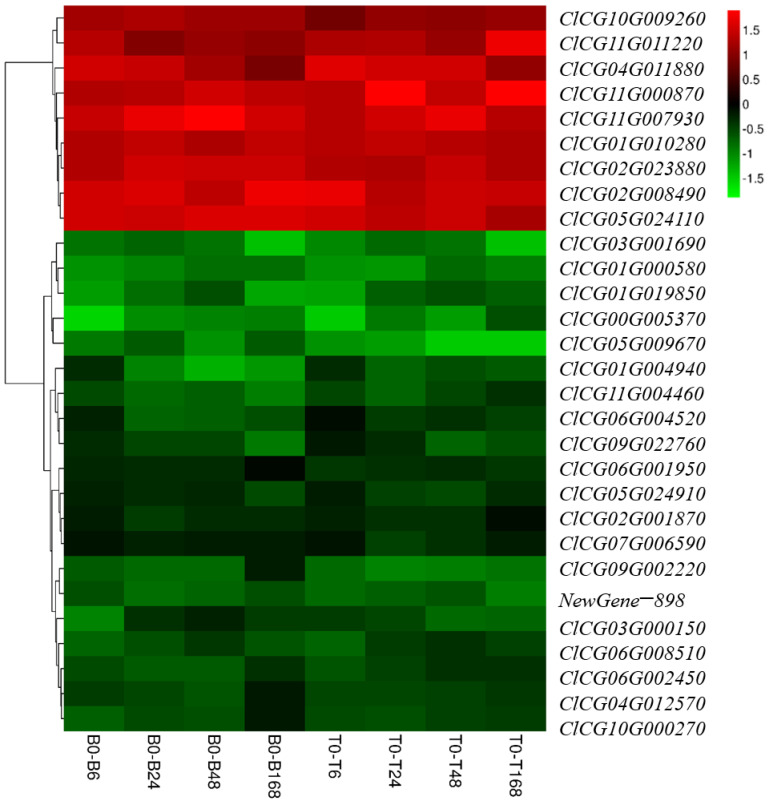
Expression level of DEGs in the glutathione metabolism pathway. B0–B6, B0–B24, B0–B48, and B0–B168 represent B0 vs. B6, B0 vs. B24, B0 vs. B48, and B0 vs. B168 in B2, respectively. T0–T6, T0–T24, T0–T48, and T0–T168 represent T0 vs. T6, T0 vs. T24, T0 vs. T48, and T0 vs. T168 in the T23 cultivar, respectively.

A total of 29 DEGs shared in the B0–B6, B0–B24, B0–B48, B0–B168, T0–T6, T0–T24, T0–T48, and T0–T168 groups, respectively (Figure 3). A total of 9 DEGs were upregulated and 19 genes were downregulated (Figure 9). We selected eight genes with the highest differential expression as salt tolerance candidate genes and preliminarily identified their functions in the yeast system.

**Figure 10:**
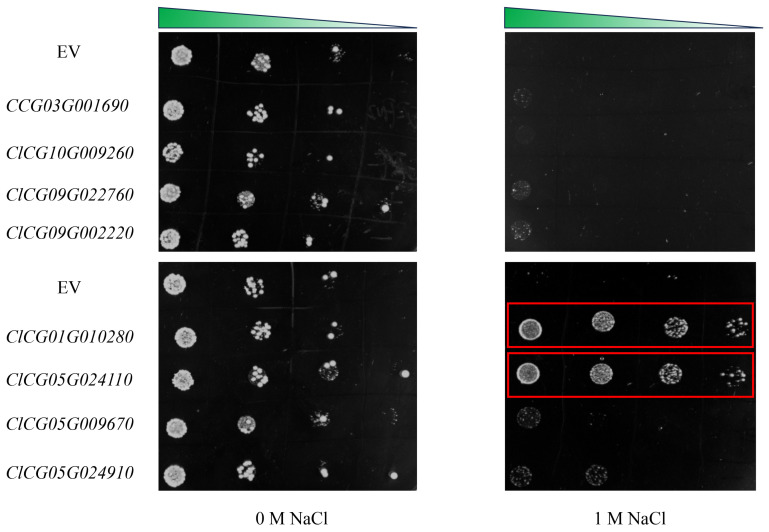
Responses to NaCl stresses through yeast dilution bioassay with the wild-type strain. Salt tolerance candidate genes transformed with pRS-416-GFP. Yeast wild-type (EV) strain and salt tolerance candidate gene-overexpressing cells were grown in URA liquid medium for 24 h at 28 °C. The cell solutions were diluted to an OD_600_ value of 0.3 and exposed to 1 M NaCl.

These genes are *ClCG03G001690* (uncharacterized protein *LOC101209654*), *ClCG10G009260* (*mitochondrial AOX2*), *ClCG09G022760* (*glyceraldehyde-3-phosphate dehydrogenase, GAPCP2*), *ClCG09G002220* (*beta-hordothionin-like*), *ClCG01G010280* (*tonoplast dicarboxylate transporter*), *ClCG05G024110* (*NAC domain-containing protein 1*), *ClCG05G009670* (*nigrin b*), and *ClCG05G024910* (*pyruvate dehydrogenase E1 component subunit alpha, mitochondrial*), respectively. We generated an overexpression model of yeast using the pRS416 vector. The yeast cells with salt tolerance candidate gene overexpression were exposed to 1 M NaCl. Under NaCl stress, *ClCG01G010280* and *ClCG05G024110* were highly tolerant when compared to EV (Figure 10). These results indicate that the *ClCG01G010280* and *ClCG05G024110* genes play an important role in response to salt stresses.

## 4. Discussion

Salt stress significantly impacts plant growth and development, notably inhibiting seed germination, root length, and plant height. In this study, watermelon plants subjected to high salt stress exhibited the dehydration of cotyledons, yellowing of leaves, and yellowing or even blackening of roots. The growth inhibition of B2 (salt-sensitive) was more pronounced compared to that of T23 (salt-tolerant). This research explores the physiological and molecular mechanisms of watermelon roots underlying the effects of salt stress.

### 4.1. Physiological Response to Salt Stress by Watermelon Seedlings

Salt stress can promote the accumulation of ROS in plant cells. Low levels of ROS can activate signaling pathways, while the excessive accumulation of ROS can damage the cell membrane structure. Lipid oxidation produces various secondary products, exacerbating oxidative damage. MDA is the main product of polyunsaturated fatty acid peroxidation and an important indicator of membrane lipid peroxidation. Some studies have reported a significant upregulation of MDA content in vegetables under salt stress [48,49,50]. Our research results indicate that the MDA content fluctuates in both genotypes of watermelon roots, possibly due to the plant’s adaptation to salt environments. In addition, there are some physiological differences in salt tolerance among different varieties. The O^2−^ content in B2 is significantly higher than that in T23. These results may be related to higher ROS scavenging activity or stronger physiological regulation of salt-tolerant T23, which enables T23 to better adapt to salt stress environments, which is consistent with previous research findings [51]. As is well known, the activity of antioxidant enzymes including SOD, POD, and CAT also increases to remove ROS from plant cells. Kordrostami et al. found that the SOD and POD activities of rice varieties with strong salt tolerance were higher or increased faster [52]. We found in this study that the SOD activity of T23 was significantly higher than that of B2, and the POD activity was higher in T23 than in T2 within 48 days after NaCl treatment. However, the CAT activity of T23 did not show a significant increase compared to B2, but rather showed the opposite trend of H_2_O_2_ changes. This may be because CAT uses H_2_O_2_ as the reaction substrate, and there is feedback regulation between the CAT activity and H_2_O_2_ content. These results indicate that T23 exhibits stronger antioxidant activity under salt stress, resulting in a lower O^2−^ content than salt-sensitive B2.

Proline accumulation is a common physiological response of plants under various abiotic stresses [53,54]. The accumulation of proline can reduce protein hydrolysis, stabilize the subcellular structure, eliminate free radicals, and increase the redox potential. Higher levels of proline can also prevent cell dehydration, maintain internal cell stability, and reduce salt stress’s harmful effects. Previous studies have shown that the proline content in watermelon increases with the duration of salt stress, and salt-tolerant varieties accumulate more proline than salt-sensitive varieties [6,33,55]. In this study, the proline content increased with the increase in the salt treatment time, and the proline content of T23 was significantly higher than that of B2. Soluble sugars (such as sucrose, glucose, and fructose) and soluble proteins regulate the cell osmotic potential through accumulation, maintain cell water balance, and alleviate salt stress damage to cells. In addition, soluble sugars can stabilize cell membranes and protein structures, preventing membrane lipid peroxidation and protein denaturation caused by salt stress. Our research found that after salt stress, the soluble sugar and soluble protein content in different genotypes of watermelon significantly increased, and the change in the soluble sugar content in T23 was greater than that in B2. This is consistent with previous experimental reports on watermelon [24,56]. These results indicate that T23 accumulates more proline and soluble sugars and has stronger antioxidant activity under salt stress, resulting in a lower O^2−^ content than that in salt-sensitive B2 and a stronger salt tolerance than that of B2.

### 4.2. Change in Gene Transcription of Watermelon Seedlings Under Salt Stress

Watermelon plants also exhibit positive physiological responses under salt stress. Here, transcriptome analysis is used to explore key salt-responsive metabolic pathways and DEGs. As the salt stress treatment time increases, the number of DEGs in T23 gradually decreases, while the number of DEGs in B2 gradually increases. This result shows that T23 can adapt to salt stress by activating numerous gene expressions. The upregulated DEGs in B2 and T23 were less than the downregulated DEGs, which is similar to the expression of DEGs in watermelon inbred line TN07011 after salt stress treatment [33]. These results collectively indicate that salt stress has a negative impact on watermelon gene transcription, leading to the downregulation of most DEGs’ expression within 48 h after salt stress treatment. The GO enrichment analysis showed differences in the enriched DEGs between the two watermelon varieties under salt stress. Four main GO enrichment terms were identified for the response of B2 and T23 to salt stress—binding, catalytic activity, transporter activity, and transcriptional regulatory factor activity—indicating that these are important pathways for watermelon plants to respond to salt stress. A previous study on GO enrichment analysis in watermelon showed that DEGs are mainly related to catalytic activity and binding [34]. In addition, Wang et al. found that most DEGs identified in watermelon are associated with transporter activity [57]. Du et al. found that 177 TF genes were detected with significantly higher expression levels in a salt-tolerant maize variety compared with a salt-sensitive variety [58]. Wang et al. reported that the analysis of differentially expressed transcription factors (TFs) suggested that WRKY TFs could contribute to the difference in salt tolerance between the two maize lines [59]. In summary, these findings indicate that binding, catalytic activity, transporter activity, and transcriptional regulatory factor activity play crucial roles in salt stress tolerance in watermelon.

The KEGG analysis further investigated the enrichment of DEGs’ metabolic pathways. Under salt stress, there are significant differences in the KEGG enrichment pathways between the two watermelon varieties. In the four experimental comparisons of B2, four metabolic pathways were enriched together, including plant hormone signaling transduction, diterpenoid biosynthesis, plant–pathogen interactions, and plant MAPK signaling pathways. Previous studies have reported that plant hormone signaling pathways play crucial roles in *aquilegia* and *Sophora alopecuroides* salt stress adaption [60,61]. Ma et al. discovered key genes involved in plant hormone signaling and mitogen-activated protein kinase signaling pathways, as well as major genes involved in proline biosynthesis and starch and sucrose metabolism, in salt-induced salt-tolerant poplar leaves [62]. Wang et al. reported that two gene modules are related to salinity, and the genes in these modules are mainly involved in plant–pathogen interactions, plant MAPK signaling pathways, and terpene biosynthesis in cotton [63]. However, in the four experimental comparisons of T23, two metabolic pathways were co-enriched, including thiamine metabolism and carbon metabolism. Fu et al. emphasized the activation of thiamine metabolism and carbon metabolism pathways in soybeans under salt stress [16]. These results indicate that watermelons of different genotypes have different ways of resisting salt stress. Meanwhile, previous studies reported that there were distinct differences in the mechanisms of response to salt stress in soybean [64], maize [65], *Brassica campestris* [66], and *Sorghum* [67]. Plant hormone signal transduction, the biosynthesis of diterpenes, interactions between plants and pathogens, and MAPK signaling pathway—plants, thiamine metabolism, and carbon metabolism play important roles in watermelon’s resistance to salt stress.

### 4.3. Salt-Tolerant Candidate Genes ClCG01G010280 and ClCG05G024110 in Watermelon

The tonoplast dicarboxylate transporter is a membrane protein found in the vacuolar membrane (tonoplast) of plant cells. It plays a crucial role in the transport of dicarboxylates, such as malate and succinate, across the tonoplast [68]. This transporter is essential for maintaining cellular homeostasis, regulating pH, and facilitating metabolic processes within the vacuole [69,70]. Furthermore, it plays a role in plant responses to environmental stresses, such as aluminum and salinity, by regulating the accumulation of organic acids in the vacuole [71,72]. In the present study, we found that the expression of the gene *ClCG01G010280* encoding the tonoplast dicarboxylate transporter is induced by salt stress. We preliminarily validated in the yeast system that the overexpression of *ClCG01G010280* can enhance the salt tolerance of yeast. These results suggest that *ClCG01G010280* may serve as a candidate gene for regulating salt tolerance in watermelons.

NAC transcription factors are a widely distributed family of transcription factors in plants, playing crucial roles in plant growth and development, stress responses (such as salt stress, drought, and low temperature), and hormone signaling. NAC transcription factors can help maintain intracellular ion homeostasis by regulating the expression of ion transporters, such as Na^+^/H^+^ antiporters. *PvNAC52* promotes Na^+^ efflux and reduces Na^+^ accumulation in the cytoplasm by regulating the expression of the *SOS1* (Salt Overly Sensitive 1) gene [73]. Additionally, NAC transcription factors can activate the expression of antioxidant enzymes, such as SOD, CAT, and POD, thereby alleviating oxidative stress. *TaNAC29* confers salt stress tolerance through reducing H_2_O_2_ accumulation and membrane damage by enhancing the antioxidant system [74]. NAC transcription factors also regulate the synthesis of osmoregulatory substances, such as proline and soluble sugars, helping to maintain cellular osmotic balance. For example, *ThNAC4* increased osmoprotectant (proline and trehalose) contents under stress conditions [75]. Furthermore, NAC transcription factors interact with plant hormone signaling pathways, such as ABA, ethylene, and JA, to participate in salt stress responses. *OsNAP* confers salt stress responses through the ABA pathway [76]. In the present study, we found that the expression of the gene *ClCG05G024110* encoding NAC is induced by salt stress. We preliminarily validated in the yeast system that the overexpression of *ClCG05G024110* can enhance the salt tolerance of yeast. These results suggest that *ClCG05G024110* may serve as a candidate gene for regulating salt tolerance in watermelons. However, the molecular and specific functional mechanisms by which *ClCG01G010280* and *ClCG05G024110* regulate watermelon salt tolerance still need further exploration.

## 5. Conclusions

Through the analysis of phenotypic, physiological parameters, transcriptomic data, and gene expression differences between the salt-tolerant watermelon variety T23 and the salt-sensitive variety B2 under 200 mM NaCl treatment, this study revealed the different response mechanisms of the two watermelon varieties to salt stress. T23 exhibited a strong salt tolerance, with significant increases in physiological indicators such as SOD, POD, and CAT activities, as well as the proline, soluble sugar, and soluble protein content, and no plant mortality was observed throughout the salt stress period. In contrast, B2 showed severe dehydration, leaf wilting, and root yellowing under salt stress, ultimately leading to the death of some plants. The transcriptomic analysis indicated significant differences in the number and expression patterns of DEGs between T23 and B2 under salt stress. T23 had fewer DEGs, mainly concentrated in the early response stage, while B2 had a higher number of DEGs, which increased significantly over time. The GO and KEGG enrichment analyses further revealed different metabolic pathways in the two varieties under salt stress. T23 enhanced salt tolerance primarily through carbon metabolism and thiamine metabolism pathways, while B2 relied on plant hormone signal transduction and MAPK signaling pathways. Additionally, the preliminary functional verification of some candidate genes (such as *ClCG01G010280* and *ClCG05G024110*) in the yeast system indicated their important roles in salt tolerance. In summary, the salt tolerance of T23 may be closely related to its more effective antioxidant system, metabolic regulation, and faster regulation of specific gene expression. These findings provide important theoretical foundations and candidate gene resources for salt-tolerant watermelon breeding.

## Data Availability

The raw sequence data reported in this paper have been deposited in the Genome Sequence Archive (Genomics, Proteomics & Bioinformatics 2021) in the National Genomics Data Center (Nucleic Acids Res 2024), China National Center for Bioinformation/Beijing Institute of Genomics, Chinese Academy of Sciences (GSA: CRA023068), and are publicly accessible at https://ngdc.cncb.ac.cn/gsa accessed on 17 February 2025.

## Supplementary Materials

The following supporting information can be downloaded at https://www.mdpi.com/article/10.3390/plants14071013/s1. Table S1: The composition of the Hoagland nutrient solution. Table S2: Primer sequences for qRT-PCR and gene cloning. Table S3: RNA-seq data quality. Table S4: Number of DEGs in comparisons of different groups.
